# Melanoma: the role of surgery in the era of new therapies

**DOI:** 10.1186/1479-5876-12-195

**Published:** 2014-07-11

**Authors:** Nicola Mozzillo, Paolo A Ascierto

**Affiliations:** 1 Department of Melanoma and Soft Tissue, Istituto Nazionale Tumori Fondazione “G. Pascale”, Via Mariano Semmola, 80131 Naples, Italy

## 

Stage IV melanoma has historically been associated with poor outcomes, with a typical one-year survival rate of around 25% [1]. In particular, patients who develop distant metastases have a poor prognosis. Treatment of these patients involves systemic medical therapy, radiotherapy and surgery.

However, systemic drug therapy as a treatment option has generally been ineffective in metastatic melanoma. Although several single-agent chemotherapies have shown activity against melanoma, complete responses are rare, with overall response rates of 8–23% and median overall survival (OS) of 6–11 months [2]. A meta-analysis of 48 studies demonstrated that the benefit from any of the various combinations of chemotherapy and biochemotherapy is about 4.2%, with a mean OS of 6 months [3].

However, recent years have seen an improvement in our understanding of the melanoma metastatic cascade, providing important new information about the molecular events that drive melanoma initiation and progression and allowing the development of novel therapies. In 2011, ipilimumab was the first new agent approved for the treatment of patients with advanced melanoma in over three decades. Ipilimumab is a fully human monoclonal antibody directed against cytotoxic T-lymphocyte-associated antigen-4 (CTLA-4), a negative regulator of T-cell-mediated immune responses. In phase III trials, treatment with ipilimumab significantly extended OS compared with control in both pre-treated and treatment-naϊve patients, and follow-up data from clinical trials suggest ipilimumab can provide durable clinical benefit and long-term survival [4,5]. The patterns of tumour response to ipilimumab differ from those observed with cytotoxic chemotherapies, since patients may have a delayed yet durable response and obtain long-term survival benefit despite initial tumour growth. Approximately 10% of patients have objective responses by standard criteria, whereas 10–20% show stable disease or minor responses that translate into a clinical benefit [6,7]. In most patients, the onset of clinical response is between weeks 12 and 24 and may persist for ≥1 year before maximal response occurs.

The first targeted therapy for advanced melanoma with a favorable impact on survival, vemurafenib, was also approved in 2011. Vemurafenib is a tyrosine kinase inhibitor of the oncogenic BRAFV600 protein kinase. Approximately 50% of melanomas harbour activating BRAF mutations, especially those patients with tumours arising on skin without chronic sun-induced damage [8]. In clinical trials, treatment with vemurafenib was associated with an OS of 77% at 6 months and 58% at 12 months, although median OS remained at 13.6 months due to disease relapses [9,10]. Other targeted therapies are also in development, either with the same molecular target (e.g. dabrafenib), or targeting steps in the pathway downstream of BRAF (e.g. MEK inhibitors). Further improvements in targeted approaches are expected from ongoing clinical trials, aiming to potentiate the activity of BRAF inhibitors through combined or sequential therapy with other molecules, both immune-based and other targeted agents [11]. These combination therapies (e.g. BRAF plus MEK inhibitors) also aim at lowering the skin toxicities observed with BRAF inhibition.

Despite the major advances offered by these new systemic therapies, surgery of stage IV melanoma remains an important therapeutic tool that can be used to rapidly and safely resolve localised disease. The rational for surgical resection as first option in stage IV melanoma is based on several factors. Single lesions are best treated by surgery while studies have shown complete resection is possible in 25% of stage IV patients (M1a through M1c inclusive) [12]. The surgical procedure has acceptable morbidity and mortality and is associated with favourable survival rates [13].

Several prognostic factors for surgery in metastatic melanoma have been identified (Table 1). In particular, patients who have limited sites of metastatic disease, prolonged disease-free survival and a tumour-volume doubling time of >60 days may be amenable to surgical resection [14-16]. It is also important to consider whether the disease burden can be completely resected. Complete surgical excision of limited metastatic disease can result in prolonged progression-free survival (PFS) in carefully selected patients. Surgery for distant metastatic melanoma, however, is rarely curative since the majority of patients with distant metastases have widespread micrometastatic disease even if clinical and imaging criteria suggest limited spread.

**Table 1:** Prognostic factors for surgical resection in stage IV melanoma

Major	Ability to achieve complete surgical resection
	Initial site of metastasis
	Number of metastatic lesions
	Tumour-volume doubling time
Minor	Disease-free interval
	Preceding stage

The rationale for considering surgery as a major component of metastatic melanoma therapy is further supported by the observation that repeat resection of recurrent distant metastatic melanoma can prolong survival and that metastases can themselves metastasize. The potential for surgery to benefit individual patients is suggested by the results of a prospective, phase II study from the Southwest Oncology Group, in which 64 of 77 carefully selected patients underwent complete resection of all sites of metastatic disease [17]. At a median follow-up of five years, the median durations of PFS and OS were 5 and 21 months, respectively. Overall, the three- and four-year survival rates were 36% and 31%, although late relapses continued to be observed after this time. Resection should be reserved for the relief or prevention of morbidity due to local tumor growth and for patients in whom a longer survival might be expected with surgical rather than medical treatment.

Large tumour masses are difficult to eradicate with systemic therapy alone and surgery in combination with novel immuno- and targeted therapies can potentially improve clinical outcomes and/or patients’ quality of life. Indeed, surgery should be offered to reduce the target of subsequent adjuvant medical treatment whenever possible.

BRAF inhibitors (vemurafenib and dabrafenib) alone and in combination (dabrafenib/trametinib, vemurafenib/cobimetinib, encorafenib/binimetinib) can have a significant clinical impact with a rapid improvement in symptoms and metabolic shut-down at PET scan assessment. However, not all metastatic sites respond to therapy and surgery may be required to remove resistant lesions. Data from a phase I study [18] and from an expanded access program [19] clearly indicate improved PFS and OS in patients with a low tumoural volume, suggesting that the use of surgery to remove resistant clones and reduce tumour burden may be beneficial. Indeed, data from a phase I trial evidenced that, for a subset of patients with disease progression, continuation of vemurafenib treatment is potentially beneficial after local therapy [20]. In this study, 18 patients who continued vemurafenib >30 days after surgery or radiotherapy at a site of disease progression had a median overall survival of over 15.5 months from initiation of BRAF inhibitor therapy. This compares with a median overall survival of 1.4 months in patients who did not continue treatment. Moreover, patients with low tumour burden seemed to have a better response to vemurafenib.

Surgery may also have an important role in combination with immunotherapy. Indeed, the removal of lesions that may be resistant to treatment with ipilimumab may improve outcomes for some patients. Pathological evaluation of the excised tissue is important to assess the presence of immune-infiltrate. In several cases, analysis of the excised tissue has revealed the presence of a diffuse immune infiltration which correlated with the outcome of these patients [21]. Thus, reduction of the tumor mass which can be obtained with surgery is important in combination with immunotherapy.

In conclusion, surgery still represents one of the four pillars of treatment for stage IV melanoma, together with radiotherapy, chemotherapy and new immunotherapy approaches.

### Competing interests

PAA received research funding from Bristol-Myers Squibb, Roche-Genentech, and Ventana. He also has/had a consultant or advisory role for Bristol-Myers Squibb, Roche-Genentech, Merck Sharp & Dohme, GlaxoSmithKline, Ventana, and Novartis. He received honoraria from Bristol-Myers Squibb, Roche-Genentech, and GlaxoSmithKline. NM has no competing interest to declare.

### Authors’ contribution

NM and PAA drafted and approved the final manuscript.
